# Demystifying the nutritional and anti-nutritional genetic divergence of Pakistani chickpea (*Cicer arietinum* L.) genetic resource via multivariate approaches

**DOI:** 10.3389/fnut.2024.1407096

**Published:** 2024-10-04

**Authors:** Saima Jameel, Amjad Hameed, Tariq Mahmud Shah, Clarice J. Coyne

**Affiliations:** ^1^Nuclear Institute for Agriculture and Biology College, Pakistan Institute of Engineering and Applied Sciences, Faisalabad, Pakistan; ^2^USDA–ARS Plant Germplasm Introduction and Testing, Washington State University, Pullman, WA, United States

**Keywords:** chickpeas, differential proteins, phytic acid, PCA, correlation

## Abstract

Chickpeas are a highly versatile functional food legume that possesses the capacity to boost human health and has the potential to alleviate malnutrition-related deficiencies. To investigate whole seed-based nutritional and anti-nutritional composition, a set of 90 chickpea genotypes (66 desi and 24 kabuli) was collected from different research organizations in Pakistan. Significant variation (Tukey HSD test, *p* < 0.05) was perceived among genotypes for traits under investigation. The genotypes, with maximum total soluble proteins (TSPs) (34.92%), crude proteins (CPs) (30.13%), and reducing sugars (17.33 mg/g s. wt.), i.e., Punjab-2000 (desi); total free amino acids (TFAs) (3.34 g/100 g DW), i.e., Wild Hybrid-15 (desi), albumins (227.67 mg/g s. wt.), i.e., Sheenghar-2000 (desi); globulins (720 g s. wt.), i.e., ICCV-96030 (desi); salt-soluble proteins (200 mg/g s. wt.), i.e., ILWC-247 (desi); total soluble sugars (TSSs) (102.63 mg/g s. wt.), i.e., CM1051/11 (desi); non-reducing sugars (95.28 mg/g s. wt.), i.e., NIAB-CH2016 (desi); starch content (83.69%), i.e., CH55/09 (kabuli); and the genotypes with least value of anti-nutritional factors glutelin (3.33 mg/g s. wt.), i.e., Wild Hybrid-9 (desi); hordein (1.38 mg/g s. wt.), i.e., Noor-2013 (kabuli); tannins (5,425 uM/g s. wt.), i.e., Wild Hybrid-1 (desi); and phytic acid (PA) (0.18 mg/g s. wt.), i.e., Bhakhar-2011 (desi), could be the promising genotypes to formulate health-promoting plant-based food products. Data were also analyzed for principal component analysis (PCA), correlation, and agglomerative hierarchical clustering. PC-1 revealed the highest contribution (20.83%) toward cumulative variability, and maximum positive factor loading was delivered by TSSs (0.85) followed by starch content (0.729). Genotypes were grouped into three distinct clusters based on high average values of traits under investigation. Cluster I encompassed genotypes with a high mean value of CP content, albumins, hordein, and glutelin; Cluster II encompassed genotypes with a high mean value of TSPs, TSSs, non-reducing sugars, globulins, salt-soluble sugars, starch, and TFAs; Cluster III encompassed genotypes with high tannins, reducing sugars, and PA. Identified desi and kabuli genotypes exhibiting superior seed quality traits and minimal anti-nutritional factors can be used in chickpea breeding programs aimed at improving seed nutritional quality in future breeding lines.

## Introduction

The world’s current population stands at approximately 6.5 billion individuals; projections indicate that this number will surge to approximately 9 billion by 2050. This impending population growth presents a mounting dilemma: how to meet the escalating demand for food with limited resources (1, 2). The relentless increase in the global population is surpassing the capacity of worldwide food production, leading to extensive food insecurity and malnutrition in numerous regions, notably across Asia, Africa, and South America (3, 4). Simultaneously, in developing countries, the dearth of high nutritional quality and nutrient-dense agricultural food commodities increases disease prevalence, particularly among the poor (5). Food insecurity arises when people are commonly anxious about their capacity to acquire a sufficient amount of nutritious, safe, affordable food (6). It is a worldwide issue and can occur because of the non-availability of healthier food choices or lack of income to afford a healthier diet by common people (7). Food insecurity and malnutrition in all their forms are more responsible for poor health than any other cause in low-income countries (8). Malnutrition is exacerbated by low consumption, a lack of diet diversity, and nutrient-deficient food. Malnutrition has been reported to reduce global gross domestic product (GDP) by 10% annually (9). It is expected that more than two billion people globally are affected by hidden hunger, and more than double the 805 million individuals do not get sufficient calories to consume (10, 11). According to research, the consumption of plant-based nutritional-rich foods is a low-risk, cost-effective intervention that May lower blood pressure, cholesterol level, and body mass index. They May also minimize the mortality rate from myocardial infarction and the number of medications required to cure chronic illnesses. Low consumption of fruits and vegetables is also one of the top 10 risk factors for mortality. Both urban and rural populations eat primarily cereal-based diets, deficient in essential minerals, which leads to poor diets and a higher frequency of nutritional deficiency illnesses (12). Serious complications, such as muscle deterioration and deformity, hindered growth and development in infants and young children, and compromised the immune system, resulting from protein deficiency. Moreover, individuals with a protein C deficiency face an elevated risk of encountering anomalous blood agglutination (13). Because of these complications, people are now more interested in “eating well” rather than simply aiming to be satiated (14). Legumes are recognized internationally as an economical and environmentally friendly alternative to meat, positioning them as the second most crucial dietary resource following cereal grains (15). The increased availability of nutritionally dense legumes, particularly for individuals in low-income areas, would play a pivotal role in addressing hidden hunger and malnutrition, ultimately contributing to the augmentation of cereal-based diets (16, 17). Legumes are referred to as “nutritional seeds for a sustainable future,” while the United Nations and Food and Agriculture Organization (FAO) have declared 2016 “The International Year of Pulses” (18).

Chickpeas (*Cicer arietinum* L.) are one of the most economically important food legumes grown worldwide because they play an important role in human nutrition (19). Enhancing the nutritional value of chickpeas and other food legumes has the ability not only to improve human health but also to fight micronutrient malnutrition deficiency (20). Cultivated chickpeas are grouped into two distinct types: desi and kabuli. The presence of anthocyanin-pigmented stem and pink flowers distinguishes features of the desi type, while the kabuli type lacks anthocyanin pigmentation and has white-colored flowers (21). It was grown to 14.84 million ha in 2021–2022, generating 18.09 million tons on an average of 1,016 kg/ha all over the world. Asia is the main chickpea-growing region in the world with an 84% production share, and Pakistan is the seventh largest chickpea-producing country in the world after India, Australia, Turkey, Ethiopia, Myanmar, and Russia (22). In Pakistan, it is mainly grown in arid/semi-arid parts of the Thal region of Punjab. These areas are completely reliant on rainfall to meet their water needs (23). In Pakistan, chickpeas are consumed in multiple forms, such as fresh green seeds, dried whole seeds, dhal, and flour, for different purposes (24). To make a variety of traditional chickpea-based products, several processing methods are being used, including roasting, frying, boiling, and puffing (18). Chickpeas are a nutrient-dense legume that encompasses a wide range of valuable nutritious components, including proteins, carbohydrates, minerals, unsaturated fatty acids, dietary fibers, vitamins, and a wide range of isoflavones (25); however, it also contains some anti-nutritional factors such as tannins and phytates that bind with proteins and minerals such as Zn and Fe (26). In comparison to other pulses, chickpeas are unique because they are a vital source of high-quality protein and carbohydrates, accounting for approximately 80% of the total dry seed mass (27, 28). Chickpeas are an imperative functional food crop as they provide not only essential nutrients but also have numerous potential health benefits against type 2 diabetes, cardiovascular disease, some cancers, and digestive diseases (29). Genetic variation and environmental conditions significantly affect the chemical composition of all crops, including cereal grains and legumes (30). Nevertheless, the advancement of quality-related research and breeding practices for chickpeas lags far behind the progress made in amplifying chickpea yield (31). To address this gap, it is crucial to prioritize the selection and breeding of cultivars that are rich in specific nutrients while also possessing the least anti-nutritional factors. Due to its expanding usage in agricultural development and the selection of appropriate genotypes for breeding crops, advances in germplasm characterization using biochemical fingerprinting have unusual advantages (32). In this perspective, the present study aimed to investigate the whole seed-based nutritional and anti-nutritional composition of chickpea genetic resources through biochemical study.

## Materials and methods

### Genetic resource

A diverse set of 90 chickpea genotypes, comprising 66 desi and 24 kabuli types, with diverse genetic backgrounds were collected from different research stations in Pakistan during 2017, and seeds were multiplied for two consecutive years (2017–2018 and 2018–2019) at the Nuclear Institute for Agriculture and Biology (NIAB), Faisalabad. Among these 66 desi genotypes, there were 25 approved Pakistani varieties, 14 advanced lines, 7 mutants, 16 wild crosses, 1 wild parent, and 3 exotic lines utilized. Overall, approximately 95 diverse parents contributed to the development of these desi lines. Regarding the Kabuli type, among the 24 Kabuli genotypes used in the present study, there were 7 approved varieties, 14 advanced lines, 2 mutants, and 1 exotic line. In total, approximately 15 diverse parents contributed to the development of these lines. The genotypes used in this study were cataloged for the year of release, institution, pedigree information, average yield, type, and other important traits, which are already cited in Table 1 of our previously published paper (33). The mature dry seeds of the germplasm, harvested during the season 2018–2019, were used for the determination of their seed-based nutritional and anti-nutritional profiling. Seed nitrogen analysis was performed at USDA-ARS Plant Germplasm Introduction and Testing, Washington State University, Pullman, WA 99164, United States.

**Table 1:** Scale for grouping chickpea genotypes in high, medium, and low categories for seed nutritional and anti-nutritional traits under investigation.

	Parameters	Low	Genotypes	Medium	Genotypes	High	Genotypes
1	**Total soluble proteins (mg/g s .wt)**	14.72–17.88	CM-88 (Desi)	18.88–32.98	CH64/11 (Kabuli)	33.17–34.92	Sheenghar-2000 (Desi)
			CH98/99 (kabuli)		Aug-242 (Desi)		CH61/09 (kabuli)
			ICCV96029 (Desi)		CH74/08 (kabuli)		Noor 2009 (kabuli)
			BKK-2174 (kabuli)		CM877/10 (kabuli)		ILWC-247 (Desi)
			CH54/07 (kabuli)		NIAB-CH2016 (Desi)		CH39/08 (Desi)
			CM3457/91 (Desi)		CH63/11 (kabuli)		CM1235/08 (kabuli)
					Paidar-91 (Desi)		CH28/07 (Desi)
					NIFA-88 (Desi)		Gocke (kabuli)
					NIFA-95 (Desi)		Punjab-2000 (Desi)
					CM2008 (kabuli)		
2	**Crude proteins (%)**	20.97–21.94	CM-2000 (kabuli)	22.50–26.97	Wild Hybrid-3 (Desi)	27.13–30.13	ICCV-96030 (Desi)
			BKK-2174 (kabuli)		Wild Hybrid-16 (Desi)		Wild Hybrid-10 (Desi)
			CH74/10 (kabuli)		Noor 2009 (kabuli)		Wild Hybrid-1 (Desi)
			CM2984/91 (Desi)		Wild Hybrid-11 (Desi)		NIAB-CH104 (Desi)
					Bittle-98 (Desi)		NIAB-CH2016 (Desi)
					CH63/11 (kabuli)		ILWC-247 (Desi)
					CM68/08 (kabuli)		CM3384/00 (Desi)
					CM2008 (kabuli)		Bittle-2016 (Desi)
					CH72/08 (kabuli)		NIFA-95 (Desi)
					Wild Hybrid-8 (Desi)		CM-72 (Desi)
3	**Total free amino acid (%)**	1.62–1.87	Tamaman-13 (kabuli)	2.00–2.97	CM98 (Desi)	3.00–3.34	CM3384/00 (Desi)
			Noor 2013 (kabuli)		CH16/06 (Desi)		CM1681/8 (Desi)
			Wanhar-2000 (Desi)		Wild Hybrid-5 (Desi)		CH61/09 (kabuli)
			Noor 2009 (kabuli)		NIFA-88 (Desi)		CH64/11 (kabuli)
					Punab2008 (Desi)		CH98/99 (kabuli)
					Wild Hybrid-6 (Desi)		Wild Hybrid-15 (Desi)
					CH60/10 (kabuli)		
					Wild Hybrid-9 (Desi)		
					NIAB-CH104 (Desi)		
					Wild Hybrid-4 (Desi)		
4	**Albumin (mg/g s .wt)**	11.50–22.67	CM-72 (Desi)	83.33–198.17	CH24/11 (Desi)	201.67–227.67	Thall-2006 (Desi)
			NIFA-88 (Desi)		CH2/11 (Desi)		Punjab-2000 (Desi)
			Paidar-91 (Desi)		CH77/08 (kabuli)		CM2008 (kabuli)
			C-44 (Desi)		CH98/99 (kabuli)		Sheenghar-2000 (Desi)
			ILWC-247 (Desi)		CH28/07 (Desi)		
			CM-2000 (kabuli)		CM68/08 (kabuli)		
					CH49/09 (Desi)		
					ICCV96029 (Desi)		
					CH54/07 (kabuli)		
					CH13/11 (Desi)		
5	**Globulin (mg/g s. wt)**	243.33–399.00	Wild Hybrid-15 (Desi)	400.33–596.00	Punjab-91 (Desi)	674.00–720.00	Wild Hybrid-13 (Desi)
			CH39/08 (Desi)		Parbat 98 (Desi)		ICCV-96030 (Desi)
			CH24/07 (Desi)		CM-72 (Desi)		
			CH74/10 (kabuli)		Wild Hybrid-3 (Desi)		
			CM2008 (kabuli)		Wild Hybrid-1 (Desi)		
			Dashat 98 (Desi)		CH74/08 (kabuli)		
			CH54/07 (kabuli)		Karak-1 (Desi)		
			Punab2008 (Desi)		Noor 2009 (kabuli)		
			CH72/08 (kabuli)		Wild Hybrid-4 (Desi)		
					Balkasar2000 (Desi)		
6	**Salt-soluble protein (mg/g s .wt)**	99.50–120.00	Wild Hybrid-1 (Desi)	120.17–187.67	CM2984/91 (Desi)	191.33–200.00	Wild Hybrid-10 (Desi)
			Noor-91 (kabuli)		CM3384/00 (Desi)		CH35/10 (Desi)
			Sheenghar-2000 (Desi)		CH49/09 (Desi)		Karak-1 (Desi)
			CM1681/8 (Desi)		NIAB-CH2016 (Desi)		CH72/08 (kabuli)
			Wild Hybrid-14 (Desi)		CH28/07 (Desi)		CH98/99 (kabuli)
			CH54/07 (kabuli)		Wild Hybrid-8 (Desi)		ICCV-96030 (Desi)
			CM98 (Desi)		CH55/09 (kabuli)		CH61/09 (kabuli)
			Wild Hybrid-15 (Desi)		Thall-2006 (Desi)		Wild Hybrid-3 (Desi)
			Wild Hybrid-11 (Desi)		CH10/11 (Desi)		Paidar-91 (Desi)
			Punjab-2000 (Desi)		Tamaman-13 (kabuli)		NIFA-88 (Desi)
7	**Hordein (mg/g s .wt)**	1.38–6.83	Noor-2013 (kabuli)	10.00–91.33	Sheenghar-2000 (Desi)	99.33–112.36	Noor 2009 (kabuli)
			Wild Hybrid-16 (Desi)		CH54/07 (kabuli)		CH56/09 (kabuli)
			CH28/07 (Desi)		Wanhar-2000 (Desi)		NIAB-CH104 (Desi)
			CM1235/08 (kabuli)		Wild Hybrid-13 (Desi)		
			CH49/09 (Desi)		Balkasar2000 (Desi)		
			CM3444/92 (Desi)		Bhakhar-2011 (Desi)		
			ILWC-247 (Desi)		ICC-4951 (Desi)		
					CH74/08 (kabuli)		
					CH13/11 (Desi)		
					Wild Hybrid-6 (Desi)		
8	**Glutelin (mg/g s .wt)**	3.33–29.33	Wild Hybrid-9 (Desi)	30.00–148.67	ILWC-247 (Desi)	154.00–203.33	CH55/09 (kabuli)
			CH49/09 (Desi)		Wild Hybrid-2 (Desi)		Wild Hybrid-1 (Desi)
			CM1051/11 (Desi)		CH56/09 (kabuli)		CH60/10 (kabuli)
			CH28/07 (Desi)		C-44 (Desi)		Noor 2009 (kabuli)
			CH35/10 (Desi)		CH74/08 (kabuli)		Wanhar-2000 (Desi)
			Noor-91 (kabuli)		CH32/10 (Desi)		CH16/06 (Desi)
			CH72/08 (kabuli)		CH40/09 (Desi)		
			Wild Hybrid-16 (Desi)		CH74/10 (kabuli)		
			NIAB-CH2016 (Desi)		CH24/11 (Desi)		
			Wild Hybrid-14 (Desi)		CM68/08 (kabuli)		
9	**Total soluble sugars (mg/g. s .wt)**	77.98–86.80	Paidar-91 (Desi)	87.23–100.84	CM2984/91 (Desi)	101.24–102.63	CH13/11 (Desi)
			Wild Hybrid-11 (Desi)		Wild Hybrid-4 (Desi)		Wild Hybrid-16 (Desi)
			CM-72 (Desi)		CH28/07 (Desi)		CH35/10 (Desi)
			CH16/06 (Desi)		Punab2008 (Desi)		ICCV-96030 (Desi)
			Noor 2009 (kabuli)		Bhakhar-2011 (Desi)		Wild Hybrid-14 (Desi)
			Wild Hybrid-5 (Desi)		Punjab-91 (Desi)		CH39/08 (Desi)
			Wild Hybrid-6 (Desi)		Wild Hybrid-1 (Desi)		CH61/09 (kabuli)
			Noor 2013 (kabuli)		Karak-2 (Desi)		CM1051/11 (Desi)
					Wild Hybrid-8 (Desi)		
					CM3457/91 (Desi)		
10	**Non-reducing sugars (mg/g. s .wt)**	64.62–76.73	Paidar-91 (Desi)	77.11–91.95	Punab2008 (Desi)	92.17–95.28	CM1235/08 (kabuli)
			CH28/07 (Desi)		Wild Hybrid-9 (Desi)		CH35/10 (Desi)
			Wild Hybrid-4 (Desi)		Noor 2009 (kabuli)		CH32/10 (Desi)
			Punjab-2000 (Desi)		Tamaman-13 (kabuli)		Parbat 98 (Desi)
					CH54/07 (kabuli)		CM1051/11 (Desi)
					Noor 2013 (kabuli)		Bittle-98 (Desi)
					CM-72 (Desi)		NIAB-CH2016 (Desi)
					Wild Hybrid-11 (Desi)		
					Wild Hybrid-6 (Desi)		
					CH16/06 (Desi)		
11	**Reducing sugars (mg/g.s .wt)**	3.59–4.59	Wild Hybrid-1 (Desi)	5.10–11.93	Wild Hybrid-10 (Desi)	12.39–17.33	CH2/11 (Desi)
			Wild Hybrid-12 (Desi)		Dashat 98 (Desi)		CH61/09 (kabuli)
			Wild Hybrid-2 (Desi)		C-44 (Desi)		CH39/08 (Desi)
			Punjab-91 (Desi)		CM2984/91 (Desi)		Paidar-91 (Desi)
			Wild Hybrid-5 (Desi)		NIFA-95 (Desi)		CH39/11 (Desi)
			Bhakhar-2011 (Desi)		Wild Hybrid-6 (Desi)		CH28/07 (Desi)
			Thall-2006 (Desi)		Wanhar-2000 (Desi)		Wild Hybrid-15 (Desi)
			Balkasar2000 (Desi)		CM3444/92 (Desi)		Punjab-2000 (Desi)
			CH64/11 (kabuli)		NIAB-CH2016 (Desi)		
			CH16/06 (Desi)		Parbat 98 (Desi)		
12	**Starch (%)**	14.13–18.15	Wild Hybrid-6 (Desi)	19.25–69.40	Wild Hybrid-4 (Desi)	70.54–83.69	Tamaman-13 (kabuli)
			Wild Hybrid-11 (Desi)		CM98 (Desi)		CH54/07 (kabuli)
			Thall-2006 (Desi)		CH77/08 (kabuli)		Wild Hybrid-15 (Desi)
			C-44 (Desi)		CM2008 (kabuli)		CH39/08 (Desi)
			Wild Hybrid-10 (Desi)		Wild Hybrid-8 (Desi)		CM407/13 (Desi)
					CM2984/91 (Desi)		BKK-2174 (kabuli)
					Wild Hybrid-5 (Desi)		ICC-4951 (Desi)
					Wild Hybrid-14 (Desi)		CH40/09 (Desi)
					CM-72 (Desi)		CH32/10 (Desi)
					Karak-2 (Desi)		CH63/11 (kabuli)
13	**Tannins (uM/g. s .wt)**	5,425–5,925	CM2008 (kabuli)	6,250–8,900	CH54/07 (kabuli)	9,050–13,775	CH74/08 (kabuli)
			NIAB-CH2016 (Desi)		Karak-2 (Desi)		CM877/10 (kabuli)
			CH98/99 (kabuli)		CH56/09 (kabuli)		CH28/07 (Desi)
			CM1235/08 (kabuli)		C-44 (Desi)		NIFA-95 (Desi)
			Dashat 98 (Desi)		CH24/11 (Desi)		Wild Hybrid-5 (Desi)
			CM3457/91 (Desi)		CH13/11 (Desi)		CH35/10 (Desi)
			Karak-1 (Desi)		Punjab-91 (Desi)		Wild Hybrid-4 (Desi)
			Thall-2006 (Desi)		CM68/08 (kabuli)		Wild Hybrid-9 (Desi)
			CH16/06 (Desi)		CH2/11 (Desi)		Wild Hybrid-7 (Desi)
			CM-88 (Desi)		ICC-4951 (Desi)		Wild Hybrid-3 (Desi)
14	**Phytic acid (mg/g s. wt)**	0.18–1.92	Bhakhar-2011 (Desi)	2.02–3.96	Wild Hybrid-11 (Desi)	4.06–6.42	CH32/10 (Desi)
			ICCV-96030 (Desi)		Wild Hybrid-4 (Desi)		CM3384/00 (Desi)
			CM1681/8 (Desi)		Wanhar-2000 (Desi)		Wild Hybrid-8 (Desi)
			NIAB-CH104 (Desi)		Bittle-2016 (Desi)		CH60/10 (kabuli)
			Wild Hybrid-7 (Desi)		Karak-2 (Desi)		CH72/08 (kabuli)
					Noor 2009 (kabuli)		CH64/11 (kabuli)
					Wild Hybrid-2 (Desi)		CH74/08 (kabuli)
					BKK-2174 (kabuli)		CM877/10 (kabuli)
					CH77/08 (kabuli)		CM3457/91 (Desi)
					Wild Hybrid-5 (Desi)		Noor 2013 (kabuli)

### Nutritional parameters

#### Sample preparation

A laboratory mini mill grinder was used to grind 20 healthy (4 to 6 g) disease-free whole seeds of each genotype into a fine powder, and to get fine flour, the material was passed through an 80 μm sieve. The fine flour was stored at room temperature in air-tight zipper bags for further analysis. Approximately 0.2 g flour sample was extracted in 2 mL of potassium phosphate buffer (50 mM) with pH 7.4. To homogenize the mixture, all the samples were vortexed and then centrifuged at 14,462 × g at 4°C for 10 min. For the determination of different biochemical analyses, the supernatant was separated and used according to different methods (34). Data were recorded in triplicate for all biochemical parameters.

#### Total soluble protein

The previously reported approach was used to estimate quantitative protein (35). Absorbance was computed at 595 nm through a spectrophotometer.

#### Seed nitrogen/crude protein

##### Seed sample preparation

Twenty healthy seeds of all genotypes were ground to a fine powder using a coffee grinder before nitrogen/protein analyses.

##### Seed nitrogen analyses and protein calculations

Seed nitrogen (N)/CP concentrations were determined using a LECO FP-528 nitrogen/CP determinator (Leco Corp., St. Joseph, MI, United States), according to the manufacturer’s instruction manual. Weighed aliquots of ethylene diamine tetra acetic acid (EDTA) were used as nitrogen standards to calibrate the instrument. Two sub-samples (0.15 g each) of each accession were analyzed for nitrogen concentration; each sample was measured two times internally in the instrument with the average reported to the operator. The two sub-sample averages were then averaged to get a nitrogen concentration value for each accession. Protein concentrations were calculated using a conversion factor of 6.25 (seed nitrogen concentration) x 6.25 (36).

### Differential proteins

#### Albumins

To obtain the protein extract, 0.02 g of ground seed flour was dissolved in 1 mL of buffer A. After 2 h of continuous stirring, the mixture was centrifuged at 4000 g for 10 min, and the supernatant corresponding to the albumin fraction was collected. The pellet was then treated with 1 mL of buffer A, centrifuged, and the supernatant was collected; repeat the same procedure and pool the collected supernatant. After mixing 5 μL of supernatant and 1 mL of Bradford dye in a cuvette, the absorbance was measured at 595 nm using a spectrophotometer (37).

#### Globulins

The albumin pellet was mixed with 1 mL of buffer B (0.01 M Tris–HCl pH 7.5 and 1 M NaCl) and stirred for 2 h before being centrifuged at 4,000 g for 10 min, twice. This fraction represents the globulin extraction. Absorbance was measured at 595 nm using a spectrophotometer with 5 μL of supernatant and 1 mL of Bradford dye (37).

#### Salt-soluble proteins

Fine ground seed samples (0.02 g) of each genotype were mixed in 2.5 mL of 0.15 M potassium phosphate buffer (pH 7.5) and 2.5 mL of 5 mM dithiothreitol (DDT), stirred for 30 min at room temperature, centrifuged at 2,000 g for 5 min, and extracted three times as before. The recovered supernatant contained salt-soluble protein. Absorbance was measured at 595 nm by adding 5 μL of salt-soluble fraction and 1 mL of Bradford dye (37).

#### Hordein

The salt-soluble protein pellet was extracted in 1% acetic acid and 2% *β*-mercaptoethanol, stirred for 0.5 h at 60°C, centrifuged at 2000 g for 5 min, and repeated three times to extract the hordein fraction. The absorbance was measured by taking 5 μL of supernatant and 1 mL of Bradford dye at 595 nm using a spectrophotometer (37).

#### Glutelin

The hordein pellet was used to extract glutelin protein fraction by adding 0.05 M sodium borate, 2% β-mercaptoethanol, and 1% sodium dodecyl sulphate (SDS). The mixture was stirred for 30 min and then centrifuged at 2000 g. The supernatant was recovered in fresh tubes three times. The absorbance was measured at 595 nm using a spectrophotometer with 5 μL of supernatant and 1 mL of Bradford dye (37).

### Sugar content

#### Total soluble sugar

TSSs were estimated by the method of Dubois et al. (38). For the determination of TSSs, extraction was performed in 80% ethanol (V/V) and the supernatant was transferred to fresh tubes. A reaction mixture was performed using 100 μL of sample and 3 mL of freshly prepared anthrone reagent in H_2_SO_4_. The mixture was heated at 97°C for 10 min and then cooled the test tubes in an ice bath. The absorbance was measured at 625 nm using a spectrophotometer.

#### Reducing and non-reducing sugars

Reducing and non-reducing sugars were determined by the method Miller (39). For the estimation of reducing sugars, 1 g of 3,5-dinitrosalicylic acid (DNS) was mixed in distilled water, followed by 30 g of sodium potassium tartrate tetrahydrate and 20 mL of 2 N NaOH, and the volume was increased to 100 mL. A reaction mixture was prepared with 200 μL of the extracted sample, 1 mL of DNS, and 1.8 mL of distilled water. Then, set it in a water bath for 15 min, cool to room temperature, and dilute with 9 mL of distilled water. The absorbance was measured at 540 nm using a spectrophotometer. Non-reducing sugars were calculated by the difference between total sugar and reducing sugars.

#### Starch content

The starch content of chickpea seed flour was determined according to the method of Malik and Srivastava (40). The fine ground seed sample (0.1 g) was extracted with 80% ethanol and centrifuged at 2900 rpm. In this method, 52% perchloric acid and anthrone solution were used, with absorbance measured at 625 nm.

### Total free amino acid (TFA)

#### Sample preparation

For the determination of TFAs, a 0.5 g of sample was weighed for all genotypes and dissolved in 10 mL of phosphate buffer (pH 7.0, 0.02 M) in 90 different tubes. The phosphate buffer (0.02 M) with pH 7.0 was prepared by adding K_2_HPO_4_ and KH_2_PO_4_ and increasing the volume to 800 mL. The pH was adjusted to 7 using di-sodium hydrogen phosphate dihydrate.

#### Assay

TFAs were determined as per Hamilton and Van Slyke (41) method. The reaction mixture was prepared by adding 1 mL of sample extract, 1 mL of 2% ninhydrin, and 1 mL of 10% pyridine solution in test tubes. This mixture was heated at 97°C for 30 min in a water bath. The samples were cooled and diluted by adding 5 mL of distilled water to each test tube. The absorbance of the colored solution was measured at 570 nm using a spectrophotometer (Hitachi, 220, Japan). The TFAs were calculated by using the following formula:


$$\begin{array}{l} \mathrm{Total amino acids}\;\left(\mathrm{\mu g}\;g-1\;\mathrm{fresh}\;\mathrm{wt}\right)=\\ \frac{\mathrm{Sample}\;\mathrm{reading}\times \;\mathrm{sample}\;\mathrm{volume}\;\times \;\mathrm{diluton}\;\mathrm{factor}}{\mathrm{Sample}\;\mathrm{weight}\;\times \;1000}\times \;100 \end{array}$$

### Anti-nutritional parameters

#### Tannins

A microcolorimetric method (42) was used to measure tannins by using the Folin–Ciocalteu (F–C) reagent. A measure of 0.05 g of seed samples was treated in 500 μL of 95% methanol. The samples were then incubated for 48 h at room temperature in the darkness. Following incubation, samples were centrifuged at 14,462 g for 5 min at room temperature. The supernatant was collected after centrifugation and mixed with 100 μL of supernatant with 100 μL of 10% (v/v) F–C reagent, then vortexed thoroughly, and then 800 μL of 700 mM Na_2_CO_3_ was added. The mixture was then placed in an incubator for 1 h. To measure tannin (0.1 g), PVPP was added to the above-prepared mixture, vortexed vigorously, and centrifuged again at 14000 g and then the absorbance was measured at 765 nm.

#### Phytic acid

Phytic acid phosphorus is determined by the modified colorimetric method described in Dhole and Reddy (43). For the estimation of phytin phosphorus, seed flour (0.05 g) was extracted in a 2.4% HCl solution, placed overnight on the shaker, centrifuged at 10,000 rpm, and the supernatant was transferred to tubes having NaCl. Salt was dissolved and incubated at-20°C for 20 min to precipitate the remaining matrix, which could interfere with the colorimetric reaction. The reaction mixture was prepared by combining 750 μL of diluted supernatant and 250 μL of modified Wade’s reagent (prepared by mixing 0.03% of FeCl_3_ and 0.3% of sulfosalicylic acid). Record the absorbance of a color reaction in a spectrophotometer at a 500 nm wavelength. The standard for sodium phytate was prepared in the range of 3–30 μg/mL.

### Statistical analysis

Statistical analysis was performed using XL-STAT software version 2014.1.02 (Copyright Addinsoft 1995–2012). To analyze and organize the resulting data, descriptive statistics were applied. Data were subjected to analysis of variance (ANOVA) with three replications. Tukey HSD test at a *p*-value of <0.05, and ANOVA was used to test the significance of the data. In the graphs, the values presented are mean ± SE. Mean data for all the traits under study was subjected to PCA. Genotype by trait biplots was created by using the first two principle components, i.e., PC-I and PC-II. Cluster analysis through agglomerative hierarchical clustering and correlation (Pearson’s test) was also used for nutritional and anti-nutritional constituents by using the same software.

## Results

### Total soluble protein (TSP)

Based on the observed differences in TSPs, desi and kabuli genotypes were assembled into three distinct groups, i.e., low, medium, and high (Table 1). In the low category, six genotypes (three kabuli and three desi types) were placed with TSP ranging from 14.72 to 17.88 g/100 g s. wt. The low category comprised 13% of kabuli and 5% of desi type of the total genotypes under investigation. The lowest value of TSP (14.72 ± 1.033 g/100 g s. wt.) was found in desi-type CM-88. Out of all tested genotypes, 75 (17 kabuli and 58 desi) genotypes were grouped in the intermediate category, with values ranging from 18.08 to 32.98 g/100 g s. wt. In this class, 70% of the genotypes were of the kabuli type, and 89% were of the desi type. In the high category, nine genotypes (4 kabuli and 5 desi) were grouped with TSP values ranging from 33.17 to 34.92 g/100 g s. wt., containing 17% kabuli and 8% desi type (Supplementary Figure S1). However, among all the tested genotypes, Punjab-2000 (desi type) depicted the highest value (34.92 ± 0.45 g/100 g s. wt.), followed by Gocke (kabuli) and the average value was 27.26 g/100 g s. wt. (Table 2).

**Table 2:** Average and range values for different studied parameters in respective desi and kabuli chickpea genotypes.

Sr#	Parameters	Range	Average	Genotype (Min value)	Genotype (Max value)
**(I)** **Nutritional components**					
1	Total soluble proteins (g/100 s. wt.)	14.72–34.92	27.26	CM-88 (D)	Punjab-2000 (D)
2	Crude proteins (g/100 g s. wt.)	20.97–30.13	24.89	CM-2000 (K)	Punjab-2000 (D)
3	Total free amino acid (g/100 g)	1.62–3.34	2.54	Tamaman-13 (K)	Wild Hybrid-15 (D)
4	Albumins (mg/g s. wt.)	11.50–227.67	139.55	CM-72 (D)	Sheenghar-2000 (D)
5	Globulins (mg/g s. wt.)	243.33–720.00	474.76	Wild Hybrid-15 (D)	ICCV-96030 (D)
6	Salt-soluble proteins (mg/g s. wt.)	99.50–200.00	150.75	Wild Hybrid-1 (D)	ILWC-247 (D)
7	Hordein (mg/g s. wt.)	1.38–112.36	38.98	Noor-2013 (K)	NIAB-CH104 (D)
8	Glutelin (mg/g s. wt)	3.33–203.33	76.49	Wild Hybrid-9 (D)	CH16/06 (D)
9	Total soluble sugars (mg/g s. wt.)	77.97–102.63	93.99	Paidar-91 (D)	CM1051/11 (D)
10	Non-reducing sugars (mg/g s. wt.)	64.61–95.28	85.95	Paidar-91 (D)	NIAB-CH2016 (D)
11	Reducing sugars (mg/g s. wt.)	3.59–17.33	8.04	Wild Hybrid-1 (D)	Punjab-2000 (D)
12	Starch content (%)	14.13–83.69	46.71	Wild Hybrid-6 (D)	CH55/09 (K)
**(II)** **Anti-nutritional components**					
13	Tannins (uM /g s. wt.)	5,425–13,775	7382.22	Wild Hybrid-1 (D)	Wild Hybrid-3 (D)
14	Phytic acid (mg/g s. wt.)	0.18–6.42	3.28	Bhakhar-2011 (D)	Tamaman-13 (K)

*D, Desi; K, Kabuli.

### Crude protein (CP)

A significant variation in seed CP content provided the basis for the classification of tested genotypes into low, medium, and high categories (Table 1). The low category consisted of four genotypes (3 kabuli and 1 desi), which accounted for 13% of the kabuli type and 2% of the desi-type genotypes used in the study. However, among these genotypes, the one with the least CP content was identified in a kabuli genotype, specifically CM-2000, with a value of 20.97 ± 0.031 g/100 g DW. The medium category consisted of 72 genotypes, with a CPCP value ranging from 22.50 to 26.97 g/100 g DW. Within this category, 15 (77%) of the genotypes belonged to the desi type, while 21 (87%) were of the kabuli type. In the high category, a total of 14 genotypes (21%) were grouped, all of which belonged to the desi type. These genotypes exhibited a CP value ranging from 27.13 to 30.13 g/100 g DW. Remarkably, the desi genotype Punjab-2000 had the highest CP content, with a value of 30.13 ± 0.063 g/100 g DW. On the whole, the average value of CP was 24.89 g/100 g DW (Table 2).

### Total free amino acid (TFA)

Based on the observed differences in seed TFAs, desi and kabuli genotypes were assembled into three distinct groups, i.e., low, medium, and high (Table 1). In the high category, there were six genotypes grouped with values ranging from 3.00 to 3.34 g/100 g DW. In the high category, 4% of genotypes belong to the desi type and 12% were of the kabuli type. In general, the highest ‘TFA’ content (3.34 ± 0.043 g/100 g DW) was detected in a desi type, i.e., Wild Hybrid-15 (Supplementary Figure S3). In the medium category, 86 genotypes with ‘TFA’ values ranging from 2.00 to 2.97 g/100 g DW were grouped. Among these, 94% of genotypes belonged to the desi type and 75% were of the kabuli type. In the low category, four genotypes were placed, with values ranging from 1.62 to 1.87 g/100 g DW. The low category represented 13% of the kabuli type and 2% of desi-type genotypes, while the least ‘TFA’ content with a value of 1.62 ± 0.004 g/100 g DW was detected in a kabuli genotype, i.e., Tamaman-13, and the overall average of TFAs was 2.54 g/100 g DW (Table 2).

### Differential protein estimation

#### Albumins

Based on the observed differences in hordein content, desi and kabuli genotypes were arranged into three classes, i.e., low, medium, and high (Table 1). In the low category, six genotypes were placed with albumin content ranging from 11.50 to 22.67 mg/g s. wt. Among these genotypes, 8% were of desi type and 4% were of kabuli type. The lowest value of albumin (11.50 ± 0.500 mg/g s. wt.) was found in desi-type CM-72. Out of all tested genotypes, 80 genotypes were grouped in the intermediate category with a value ranging from 83.33 to 198.167 mg/g s. wt. In this class, 92% of the genotypes were of the kabuli type and 88% were of the desi type. In the high category, four genotypes were grouped with values ranging from 201.66 to 227.66 mg/g s. wt. Among these genotypes, 4% were of kabuli type and 4% were of desi type (Supplementary Figure S4). However, among all the tested genotypes, sheenghar-2000 (desi type) showed the highest value (227.67 ± 1.333 mg/g s. wt.) for seed albumin content and the average value was 139.55 mg/g s. wt. (Table 2).

### Globulins

A significant variation in seed globulins provided the base for the categorization of tested genotypes in low, medium, and high groups (Table 1). In the high category, there were two genotypes grouped with values ranging from 674 to 720 mg/g s. wt., and both were of desi type, accounting for 3% of total desi-type genotypes used in the study (Supplementary Figure S5). The highest seed globulins (720 ± 4.66 mg/g s. wt.) were observed in a desi type, i.e., ICCV-96030. In the intermediate category, 79 genotypes with globulins ranged from 400.33 to 596 mg/g s. wt. were categorized. Among these, 83% of genotypes belonged to the kabuli type and 89% were of the desi type. In the low category, nine genotypes were found, with values ranging from 243.33 to 399 mg/g s. wt. In the low category, 8% desi-type and 17% of kabuli-type genotypes were grouped. However, the lowest globulins with a value (243.33 ± 1.66 mg/g s. wt.) were detected in a desi-type genotype, i.e., wild hybrid-15, and the average value was 474.76 mg/g s. wt. (Table 2).

### Salt-soluble proteins

Based on the observed differences in the studied parameters, desi and kabuli genotypes were placed into three sets, i.e., low, medium, and high (Table 1). In the low category, 18 genotypes were placed with salt-soluble proteins ranging from 99.50 to 120 mg/g s. wt. Among these genotypes, 21% were of desi type and 17% were of the kabuli type. The lowest value of salt-soluble protein (99.50 ± 3 mg/g s. wt.) was found in desi-type Wild Hybrid-1. Out of all tested genotypes, 61 genotypes were grouped in the intermediate category with a value ranging from 120.167 to 187.67 mg/g s. wt. In this class, 71% of the genotypes were of the kabuli type and 67% were of the desi type. In the high category, 11 genotypes were grouped with values ranging from 191.33 to 200 mg/g s. wt. Among these genotypes, 12% were of kabuli type and 12% were of desi type (Supplementary Figure S6). However, among all the tested genotypes, ILWC-247 (desi type) showed the highest value (200.00 ± 6.33 mg/g s. wt.) for seed salt-soluble proteins, and the overall average value was 150.75 mg/g s. wt. (Table 2).

### Hordein

A significant variation in seed Hordein provided the basis for the categorization of tested genotypes in low, medium, and high groups (Table 1). In the high category, there were three genotypes grouped with values ranging from 132.33 to 236.33 mg/g s. wt. Among these two genotypes, one belongs to the kabuli type and the other belongs to the desi type, accounting for 2% of the desi type and 8% of the kabuli genotypes used in the study (Figure 3) 0.27. The highest seed hordein content (236.33 ± 9 mg/g s. wt.) was observed in a desi type, i.e., NIAB-CH104. In the intermediate category, 80 genotypes with hordein values ranging from 10 to 91.33 (mg/g s. wt.) were grouped. Among these, 84% of genotypes belonged to the kabuli type and 90% were of the desi type. In the low category, seven genotypes were found, with values ranging from 1.38 to 6.83 mg/g s. wt. (Supplementary Figure S7). In the low category, 8% of desi-type and 8% of kabuli-type genotypes were grouped. However, the lowest hordein protein content with a value of 1.38 ± 0.027 mg/g s. wt. was detected in a kabuli type genotype, i.e., Noor 2013, the overall average value was 38.98 mg/g s. wt. (Table 2).

### Glutelin

Based on the observed differences in glutelin content, desi and kabuli genotypes were arranged into three categories, i.e., low, medium, and high (Table 1). In the low category, 16 genotypes were placed with glutelin content ranging from 3.33 to 29.33 mg/g s. wt. Among these genotypes, 17% were of desi type and 21% were of kabuli type. The lowest value of glutelin (3.33 ± 0.67 mg/g s. wt.) was found in desi-type Wild Hbrid-9. Out of all tested genotypes, 69 genotypes were grouped in the intermediate category with a value ranging from 30 to 148.67 mg/g s. wt. In this class, 67% of the genotypes were of the kabuli type and 79% were of the desi type. In the high category, six genotypes were grouped with values ranging from 154 to 203.33 mg/g s. wt. Among these genotypes, 12% were of kabuli type and 4% were of desi type (Supplementary Figure S8). However, among all the tested genotypes, CH16/06 (desi type) showed the highest value (203.33 ± 4 mg/g s. wt.) for seed glutelin content, and the overall average value was 76.49 mg/g s. wt. (Table 2).

### Sugar contents

#### Total soluble sugars (TSS)

Based on the observed differences in the studied parameter, desi and kabuli genotypes were assembled into three groups, i.e., low, medium, and high (Table 1). In the low category, 13 genotypes were placed with TSSs ranging from 77.97 to 87.91 mg/g s. wt. Among these genotypes, 17% were of desi type and 8% were of kabuli type. The lowest value of TSS (77.97 ± 0.004 mg/g s. wt.) was found in desi-type Paidar-91. Out of all tested genotypes, 64 genotypes were grouped in the intermediate category with a value ranging from 88.034 to 99.72 mg/g s. wt. In this class, 79% of the genotypes were of the kabuli type and 68% were of the desi type. In the high category, 13 genotypes were grouped with values ranging from 100.41 to 102.63 mg/g s. wt. Among these genotypes, 13% were of kabuli type and 15% were of desi type (Supplementary Figure S9). However, among all the tested genotypes, CM1051/11 (desi type) depicted the highest value (102.63 ± 0.005 mg/g s. wt.) for seed TTS, while the overall observed average value was 93.99 mg/g s. wt. (Table 2).

#### Non-reducing sugars (NRS)

A significant variation in seed non-reducing sugars (NRS) provided the base for the categorization of tested genotypes in low, medium, and high groups (Table 1). In the high category, there were seven genotypes grouped with values ranging from 92.16 to 95.28 mg/g s. wt. Among these, one genotype belongs to the kabuli type and six were of the desi type, accounting for 9% of desi and 4% of kabuli genotypes used in the study (Supplementary Figure S10). The highest seed NRS (95.28 ± 0.013 mg/g s. wt.) was detected in a desi type, i.e., NIAB-CH2016. In the intermediate category, 79 genotypes with NRS values ranging from 77.11 to 91.95 mg/g s wt. were grouped. Among these, 96% of genotypes belonged to the kabuli type and 85% were of the desi type. In the low category, 13 genotypes were placed, with values ranging from 64.61 to 76.73 mg/g s. wt. In the low category, 17% desi-type and 8% of kabuli-type genotypes were grouped. However, the lowest NRS with a value (64.61 ± 0.015 mg/g s. wt.) was detected in a desi-type genotype, i.e., Paidar-91, and the overall average value of NRS was 85.95 mg/g s. wt., illustrated in Table 2.

#### Reducing sugars (RS)

Based on the observed differences in the studied parameter, desi and kabuli genotypes were organized into three sections, i.e., low, medium, and high (Table 1). In the low category, 13 genotypes were placed with reducing sugars (RS) ranging from 3.59 to 4.58 mg/g s. wt. Among these genotypes, 15% were of desi type and 4% were of kabuli type. The lowest value of RS (3.59 ± 0.008 mg/g s. wt.) was found in desi-type Wild Hybrid-1. Out of all tested genotypes, 71 genotypes were grouped in the intermediate category with a value ranging from 5.09 to 11.93 mg/g s. wt. In this group, 92% of the genotypes were of kabuli type and 74% were of desi type. In the high category, eight genotypes were placed with values ranging from 12.38 to 17.33 mg/g s. wt. Among these genotypes, 4% were of kabuli type and 11% were of desi type (Supplementary Figure S11). However, among all the tested genotypes, Punjab-2000 (desi type) depicted the highest value (17.33 ± 0.100 mg/g s. wt.) for seed RS. On the whole, the average value of RS was 8.04 mg/g s. wt. (Table 2).

### Starch content

A significant variation in seed starch content provided the basis for the categorization of tested genotypes in low, medium, and high groups (Table 1). In the high category, there were 16 genotypes grouped with values ranging from 70.53 to 83.69%. Among these, 25% were of kabuli type and desi type, accounting for 15% of the total desi-type genotypes used in the study (Supplementary Figure S12). The highest seed starch content (83.69 ± 0.50%) was observed in a kabuli-type genotype, i.e., CH55/09. In the intermediate category, 69 genotypes with starch values ranging from 19.25 to 69.40% were grouped. Among these, 75% of genotypes belonged to the kabuli type and 77% were of the desi type. In the low category, five genotypes were found, with values ranging from 14.13 to 18.15%, all were of desi type, accounting for 8% of desi genotypes used in the study. On the whole, the lowest starch content (14.13 ± 0.019%) was observed in desi type, i.e., Wild Hybrid-6, and the overall average value of starch was 46.71% (Table 2).

### Anti-nutritional parameters

#### Tannins

Based on the observed differences in the studied parameter, desi and kabuli genotypes were placed into three sets, i.e., low, medium, and high (Table 1). In the low category, 12 genotypes were placed with Tannins ranging from 5,425 to 5,925 uM /g s. wt. Among these genotypes, 12% were of desi type and 17% were of kabuli type. The lowest value of Tannins (5,425 ± 25 uM /g s. wt.) was found in desi-type Wild Hybrid-1. Out of all tested genotypes, 68 genotypes were grouped in the intermediate category with a value ranging from 6,250 to 8,900 uM /g s. wt. In this group, 75% of the genotypes were of the kabuli type and 76% were of the desi type. In the high category, 10 genotypes were placed with values ranging from 9,050 to 13,775 uM /g s. wt. Among these genotypes, 8% were of kabuli type and 12% were of desi type (Supplementary Figure S13). However, among all the tested genotypes, Wild Hybrid-3 (desi type) depicted the highest value (13,775 ± 425 uM /g s. wt.) for seed Tannins. On the whole, 7382.22 uM/g s. wt., Tannins were found in genetic resources under investigation (Table 2).

#### Phytic acid (PA)

A significant variation in seed PA content provided the base for the categorization of tested genotypes in low, medium, and high groups (Table 1). In the high category, there were 21 genotypes grouped with values ranging from 4.06 to 6.42 mg/g s. wt. In the high category, 21% of desi-type and 29% of kabuli-type genotypes were grouped (Supplementary Figure S14). Overall, the highest seed PA content (6.42 ± 0.29 mg/g s. wt.) was detected in a Kabuli type, i.e., Tamaman-13. In the intermediate category, 64 genotypes with PA values ranging from 2.02 to 3.96 mg/g s. wt. were grouped. Among these, 71% of genotypes belonged to the kabuli type and 71% were of the desi type. In the low category, five genotypes were placed, with values ranging from 0.18 to 1.92 mg/g s. wt. In the low category, all the genotypes were of desi type, accounting for 8% of desi genotypes used in the present study. However, the lowest PA with value (0.18 ± 0.015 mg/g s. wt.) was detected in a desi-type genotype, i.e., Bhakhar-2011. The overall average value of PA was 3.28 mg/g s. wt. (Table 2).

### Agglomerative hierarchical clustering analysis

**Figure 1:**
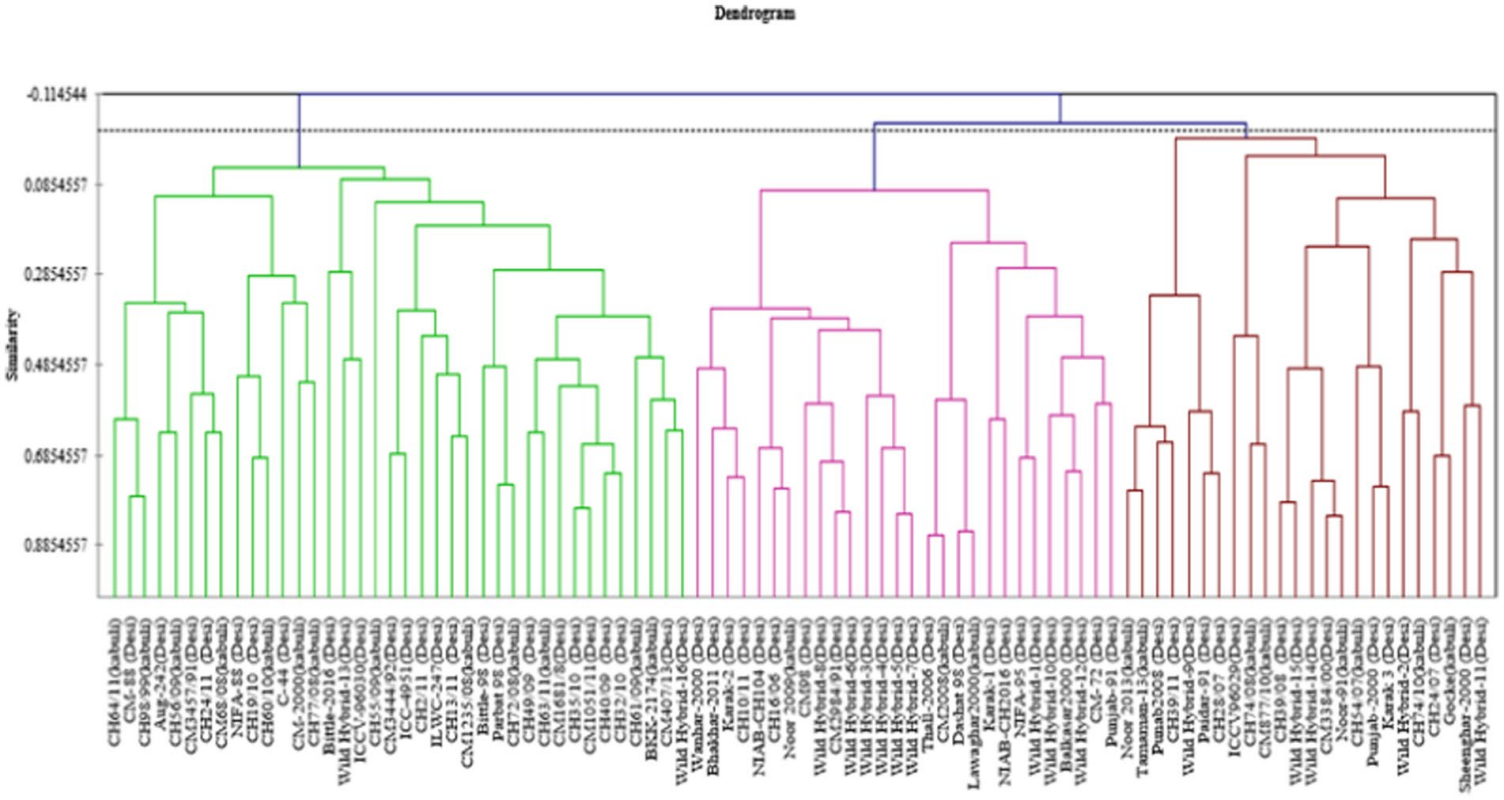
Tree diagram based on seed nutritional and anti-nutritional constituents for chickpea genotypes used in study.

Data for 14 nutritional and anti-nutritional traits along with experimental genotypes were subjected to agglomerative hierarchal cluster analysis, and a dendrogram was generated to visually inspect the clusters of genotypes. Cluster analysis cleaved 90 genotypes (desi, kabuli) into three distinct groups shown in Figure 1. Cluster I encompassed the genotypes with high mean values of CP contents, albumins, hordein, and glutelin. Cluster II contained the genotypes with high mean values of TSPs, TSSs, non-reducing sugars, globulins, salt-soluble proteins, starch, and TFAs. The genotypes with high mean values of tannins, reducing sugars, and PA were assembled in Cluster III (Table 3). The composition of clusters demonstrated that the largest cluster was II, which contained 38 genotypes, including 25 (38%) desi and 13 (54%) kabuli type, followed by Cluster I, which retained 28 genotypes consisting of 25 (38%) desi and 3 (13%) of kabuli type. Cluster III acquired 24 genotypes, including 16 (24%) desi and 8 (33%) kabuli type (Table 4).

**Table 3:** Mean values of seed nutritional and anti-nutritional constituent of chickpea genotypes in cluster analysis.

Class	Tannins	total soluble protein	crude proteins	Total soluble sugars	Non-reducing sugars	Reducing sugars	Albumins	Globulins	Salt-soluble proteins	Hordein	Gluten	Phytic acid	Starch	TFA
1	7330.357	270.714	**25.147**	90.184	84.556	5.628	**158.190**	458.524	154.875	**49.857**	**117.095**	3.036	30.776	2.387
2	7081.579	**277.136**	24.607	**97.600**	**89.092**	8.507	120.601	**510.202**	**159.636**	39.199	62.039	3.322	**58.827**	**2.633**
3	**7918.750**	267.472	25.056	92.725	82.592	**10.132**	147.806	437.583	131.861	35.516	51.701	**3.488**	46.116	2.582

Values in bold font showed the highest mean value of respective traits.

**Table 4:** Distribution of chickpea (desi and kabuli) genotypes in different clusters based on seed nutritional and anti-nutritional constituents.

Clusters	No of genotypes	Genotypes
I	28	**25 Desi** (CM-72, Karak-1, Punjab-91, NIFA-95, CM98, Balkasar-2000, Wanhar-2000, Dashat-98, Karak-2, Thall-2006, Bhakhar-2011, NIAB-CH2016, NIAB-CH104, CH10/11, CH16/06, CM2984/91, Wild Hybrid-1, Wild Hybrid-3, Wild Hybrid-4, Wild Hybrid-5, Wild Hybrid-6, Wild Hybrid-7, Wild Hybrid-8, Wild Hybrid-10, Wild Hybrid-12)**: 3 Kabuli** (Lawaghar-2000, CM-2008, Noor-2009)
II	38	**25 Desi** (C-44, NIFA-88, CM-88, Bittle-98, Parbat-98, Bittle-2016, CH40/09, CH49/09, CH32/10, CH35/10, CH2/11, CH13/1, CH24/11, CH19/10, CM1051/11, CM1681/8, CM407/13, CM3444/92, CM3457/91, Wild Hybrid-13, Wild Hybrid-16, Aug-242, ICCV-96030, ICC-4951, ILWC-247)**: 13 Kabuli** (CM-2000, CH77/08, CH55/09, CH56/09, CH61/09, BKK-2174, CH63/11, CH64/11, CM68/08, CH60/10, CH98/99, CH72/08, CM1235/08)
III	24	**16 Desi** (Paidar-91, Sheenghar-2000, Punjab-2000, Punjab-2008, Karak-3, CH28/07, CH39/08, CH39/11, CH24/07, CM3384/00, Wild Hybrid-2, Wild Hybrid-9, Wild Hybrid-11, Wild Hybrid-14, Wild Hybrid-15, ICCV-96029)**: 8 Kabuli** (Noor-91, Noor-2013, Tamaman-13, CH74/08, CH74/10, CH54/07, CM877/10, Gocke)

### Principal component analysis (PCA)

#### Seed nutritional and anti-nutritional properties

PCA is a non-parametric, simple method for mining imperative information related to confusing data sets. In the present investigation, PCA analysis was conducted for 14 seed nutritional and anti-nutritional traits of chickpea seeds. The objective of PCA is to pinpoint the minimum possible number of principal components, which can elucidate the maximum variation based on the PC scores.

**Figure 2:**
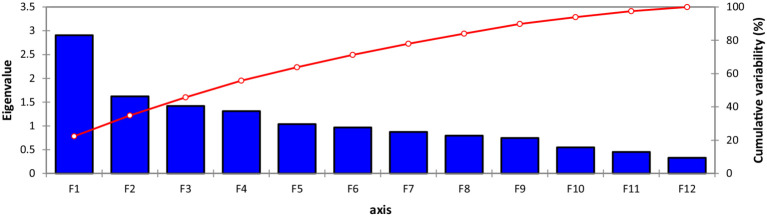
Scree plot of seed nutritional and anti-nutritional attributes representing variability.

A scree plot was also created, which is a graphical approach to anticipate the extent of the variability accompanying each one of the components extracted in a PCA (Figure 2). Out of 13 Principal component (PCs), only 6 PCs presented more than 1.0 Eigenvalue; consequently, these 6 PCs were given due importance in the present study for further explanation because the remaining PCs were not contributing significant variations (Table 4). Individually, PC-I rendered 20.83% of the cumulative variability, while PC-II, PC-III, PC-IV, PC-V, and PC-VI elucidated 11.82, 10.24, 9.52, 8.22, and 7.35% of the variability, respectively. Cumulatively, these six PCs described 68.01% of the total variability among the traits. PC-I showed positive factor loading with 10 traits including tannins, TSPs, CPs, TSSs, non-reducing sugars, reducing sugars, globulins, PA, starch, and TFAs, while the maximum contribution was delivered by TSSs (0.859), followed by starch content (0.729). It was observed that in PC-I, positive factor loading (0.072) was given by kabuli-type genotypes, while desi types showed negative factor loading (−0.072). In the case of PC-II, eight parameters represented positive factor loading, which was TSSs, non-reducing sugars, albumins, globulins, salt-soluble proteins, hordein, glutelin, and TFAs, while maximum contribution was rendered by non-reducing sugars (0.733), followed by TSSs (0.299) and anti-nutritional traits such as tannins and PA negative factor loading. It was observed that in PC-II positive factor loading (0.067) was given by the kabuli type, while desi types showed negative factor loading (−0.065). PC-III revealed positive factor loading with nine traits, including tannins, TSPs, CPs, reducing sugars, globulins, salt-soluble proteins, hordein, glutelin, and starch; however, the highest positive contribution was given by salt-soluble proteins (0.693), followed by globulin (0.433). In the case of PC-IV 8 traits showed positive factor loading, which were tannins, TSPs, TSSs, non-reducing sugars, reducing sugars, albumins, globulins, and hordein; remaining traits denoted negative factor loading; however, maximum participation was given by albumins (0.626), followed by tannins (0.471). In PC-V, positive factor loading was shown by 10 traits, including TSPs, CPs, non-reducing sugars, TSSs, albumins, globulins, salt-soluble proteins, glutelin, starch, and PA; however, the highest positive factor loading was given by TSPs (0.636), followed by PA (0.322). In the case of Cluster VI, positive factor loading was given by eight traits, including CPs, TSSs, reducing sugars, albumins, globulins, hordein, glutelin, and TFAs; moreover, maximum participation was given by CPs (0.839), followed by albumins (0.301) (Supplementary Table S1). To study the interaction between genotypes and traits under investigation, a genotype-by-trait biplot was created.

The first two principal components (PC-I and PC-II), which accounted for 32.66% of the total variability, were used for the construction of a genotype by trait biplot. In the biplot, along the x-axis, the PC-I score was plotted, and along the *y*-axis, the PC-II score was plotted along with all experimental genotypes. To understand the interrelationship of studied traits, a vector line for all traits is drawn from the origin. The knowledge about the angles between the trait vectors is deployed for interpreting the relationship of traits. An angle of 180 degrees denotes a negative connection, an angle of 90 degrees indicates a weak correlation, and a smaller angle indicates a stronger positive correlation. Through this approach, we were able to group accessions according to their relationships and defining traits.

**Figure 3:**
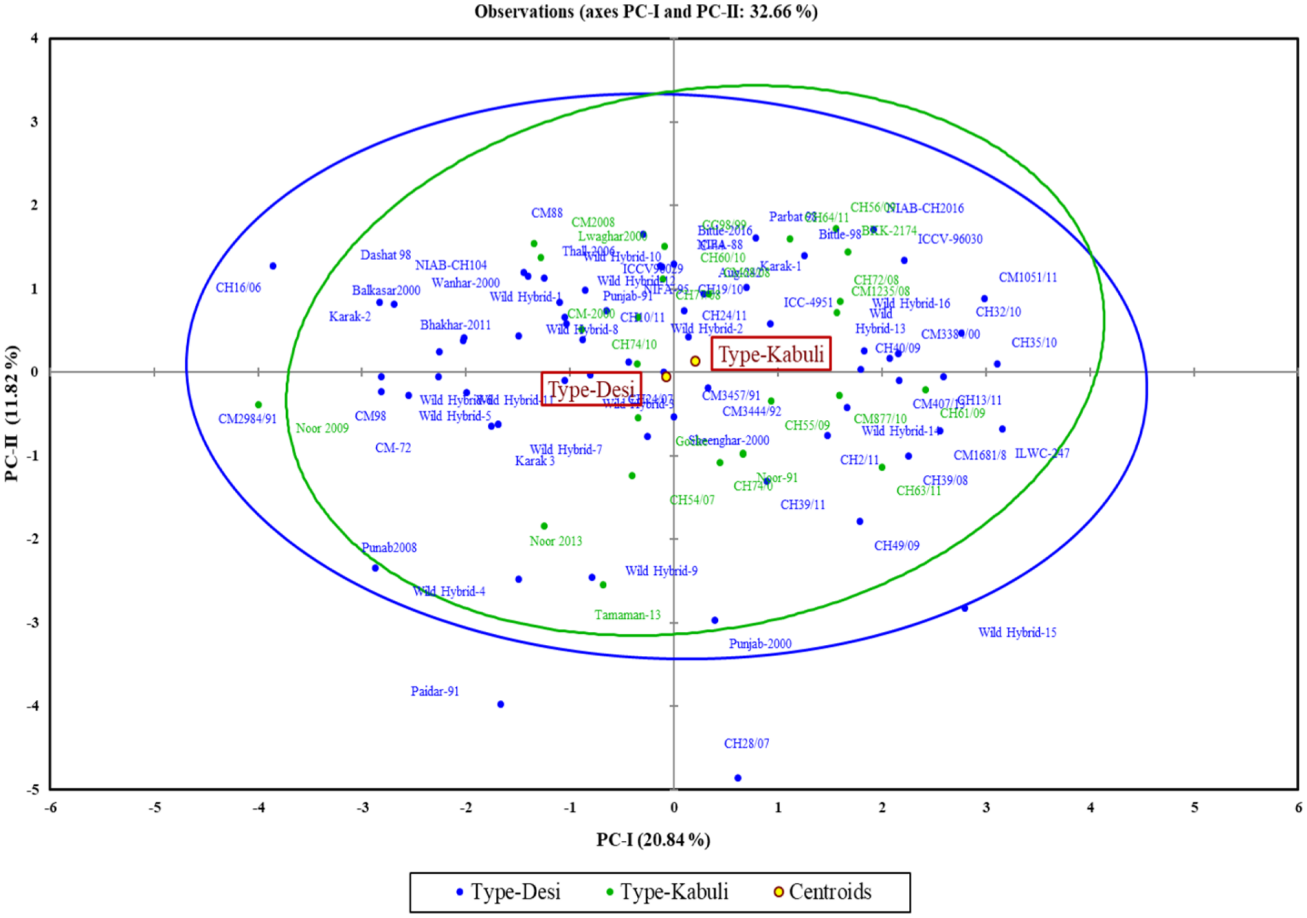
Distribution of desi and kabuli genotypes in biplot for first two principal components.

**Figure 4:**
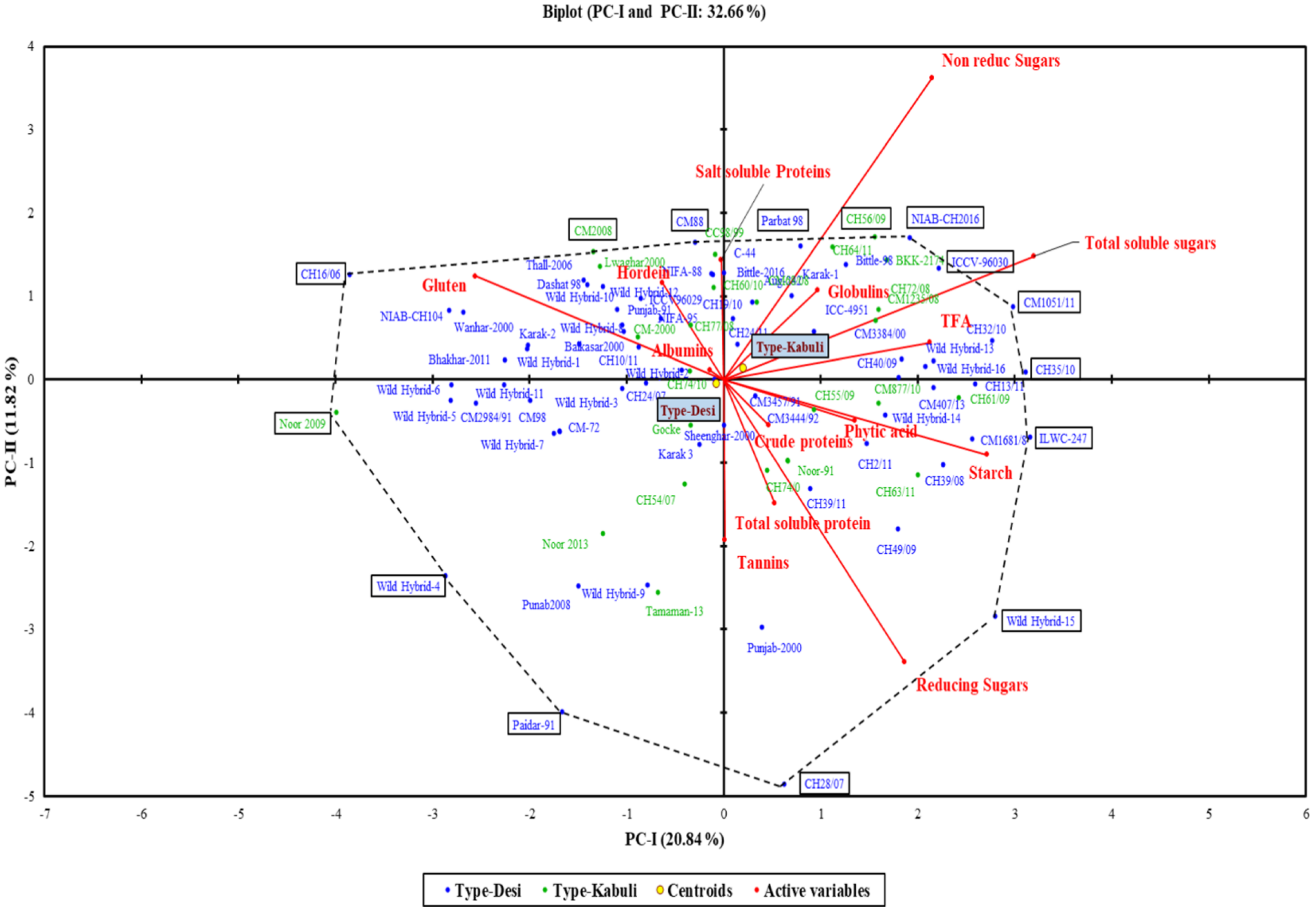
Biplot of chickpea genotypes for PC-I and PC-II.

The polygon view of the biplot assisted in recognizing the genotypes with high positive or negative values for one or more traits (Figure 3). Additionally, it was observed that the desi and kabuli genotypes were widely dispersed across all four quadrants, indicating significant genetic divergence for the traits under investigation (Figure 4).

In the PCA biplot, desi and kabuli genotypes located at or near the vertices of the polygon demonstrate significant factor scores. For PC-I, genotypes such as CH35/10, ILWC-247, Wild Hybrid-15, CH1051/11, ICCV-96030, and CH56/09 exhibited high positive factor scores. Conversely, genotypes such as Noor 2009, CH16/06, Wild Hybrid-4, Paidar-91, and Lawaghar show negative factor scores. Similarly, for PC-II, genotypes such as CM-88, Parbat 98, C-44, CH16/06, NIAB-CH2016, ICCV-98030, and CC98/99 have high positive scores. In contrast, genotypes including CH28/07, Paidar-91, CH28/07, WH-4, WH-9, and Noor-91 display negative factor scores. Furthermore, genotypes far from the origin demonstrated more variability for traits under study. While those are near each other, the origin of the biplot can be considered as genotypes with little or no variations concerning traits under investigation.

### Correlation matrix

**Figure 5:**
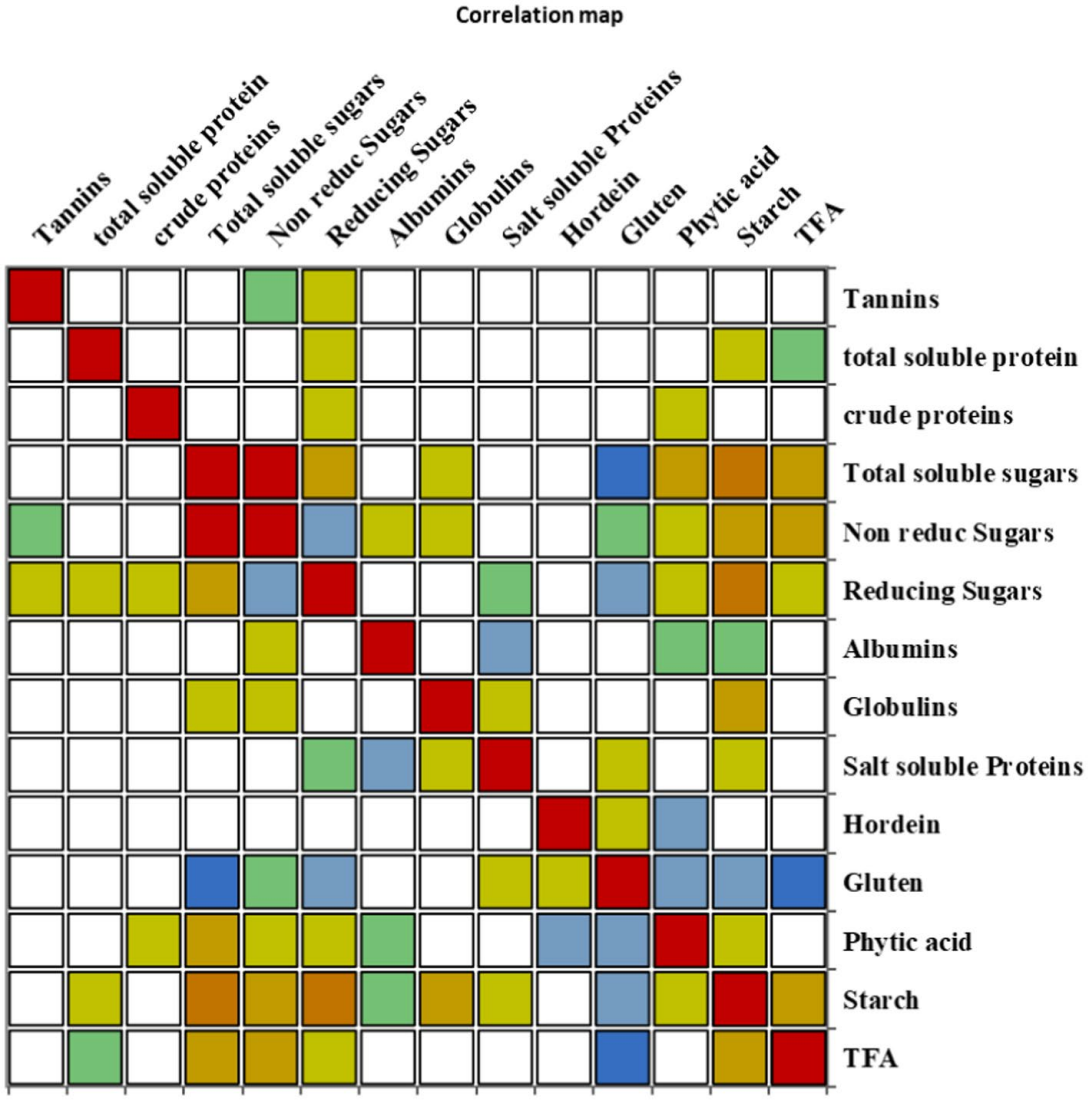
Correlation matrix showing Pearson’s correlation among nutritional and anti-nutritional traits, in chickpea genotypes.

The correlation coefficient was carried out to retrieve information about the relationship between the traits under investigation (Figure 5). Tannins, TSPs, and CP content represented no significant correlation with all other traits. TSSs depicted a significant positive correlation with non-reducing sugars, reducing sugars, PA, starch content, and TFAs; however, it revealed a significant negative correlation with gluten. Non-reducing sugars showed a significant positive association with TSSs and TFAs, while they had a significant negative association with reducing sugars. Reducing sugars had a significant positive correlation with TSSs and starch content, though it negatively correlated with non-reducing sugars and gluten content. A significant negative correlation was observed between albumin and salt-soluble proteins; however, a significant positive correlation was found between globulins and starch content. Salt-soluble protein revealed a significant negative correlation with albumin content, while a significant negative correlation was found between hordein and PA. Gluten content exhibited a significant negative association with TSSs, reducing sugars, PA, starch content, and TFAs. PA revealed a significant positive correlation with TSSs, and it had a significant negative association with hordein and gluten content. A significant positive association was revealed by starch content with TSSs, reducing sugars, globulins, and TFAs, while it had a significant negative association with gluten content. TFAs exhibited a significant positive correlation with TSSs, non-reducing sugars, and starch, and they depicted a significant negative correlation with gluten content (Supplementary Table S2).

## Discussion

Unhealthy diet and chronic diseases are the chief causes of two-thirds of global deaths. According to the WHO report, the four key metabolic changes, including obesity, high blood pressure, hyperglycemia, and hyperlipidemia, are linked with chronic disease, and they intensify the chance of death (44). To devitalize the aforementioned health risk factors, a proper and balanced plant-based nutritious diet including legumes, vegetables, and fruits could be the best option (45). For meeting the human nutritional prerequisite, the bioavailability and digestibility of plant protein are the key factors mainly when looking for substitutes for animal-based proteins (46). Legume seeds possess great nutritional value and are considered an incredible source of protein, carbohydrates, fiber, minerals, and other bioactive compounds and are poor in fats (47).

Chickpea seed-based diets are well known for their low hypersensitivity and rich nutritional package (protein, carbohydrates, minerals, vitamins, etc.), and their protein quality is considered superior to that of other legumes (25, 48). In order to address the issues of global food security and malnutrition, dedicated efforts are needed to amplify the nutritional potential of crops such as chickpeas (49).

To get in-depth biochemical insights into a diverse set (desi: kabuli) of chickpea genetic resources, seed-based nutritional, anti-nutritional, and mineral constituents were inspected in the current study. Protein represents an imperious component of chickpea seeds; its composition describes their application in different food products (50). Findings showed that the highest TSPs (34.92%) and CPs (30.13%) were detected in Punjab-2000, which belongs to the desi type, followed by the kabuli genotype Gocke. A previous report suggested that sheenghar-2000 exhibited 34.06% and Thall-2006 exhibited 23% TSP. The present findings were in the previous range for these genotypes (32). It was noticed that chickpea protein depicts good functional properties, such as oil and water absorbing capacity, solubility, foaming, emulsifying, and gelling properties, which give excellent baking characteristics (51). The range of CPs observed in the present study was 20.97–30.13%. Other studies reported that, in chickpea seeds, the maximum CPs was 30.5 and 26% noticed in desi-type chickpeas, with similar results observed in the current study (52). Another report supports our findings in which the average CPs in kabuli and desi varied from 26.7 to18.2% (53).

In the central metabolism of the seeds, amino acids play an important role. They are principally used for the synthesis of seed storage proteins, but also act as a source of energy and assist as precursors for the biosynthesis of secondary metabolites. Free amino acids are the class of amino acids that are not bound in proteins (54). The highest TFAs with a value of 3.34% was detected in a desi-type genotype, Wild Hybrid-15. In earlier research, the maximum TFAs in chickpea rhizobium-inoculated seeds of Pusa-362 was 2.65% observed, which is 0.69% less than the present finding (55). Chickpea seed storage proteins are mainly composed of albumin, globulins, glutelin, residual protein, and prolamin, and they play an important role in human nutrition and plant reproduction as well (56, 57). Albumin is a most important transporter of amino acids and a storage reservoir of protein; because of their solubility in water, they are unique (58). Present findings illustrated that maximum albumin content (22.76%) was detected in sheenghar-2000 (desi type). Previously it was reported that albumin content was 8–12%, they play an indispensable role in chickpea nutrition and growth by supplying essential amino acids (59). Globulins are salt-soluble fractions of the protein, mainly composed of vicilin and legumin; in chickpeas major, globulin is legumin and hexameric proteins (60). A desi-type ICCV-96030 showed the highest globulin content (72%). In the previous report, it was noticed that chickpea seed protein was comprised of 56% globulin, which was 16% lower than the present findings (61). Maximum salt-soluble protein (20%) was noticed in ILWC-247 (desi type). In a previous study, it was observed that 16.24% salt-soluble protein was found in wheat (34); however, the present study revealed 4% higher salt-soluble protein in chickpeas than already reported in wheat grain. Hordein, a prolamin alcohol-soluble glycoprotein found in barley and other food crops, is a concern for some individuals with gluten intolerance or celiac disease. Because of gluten intolerance or celiac disease (CD), some people are sensitive to hordein intake, so foods with low hordein content are recommended (62). Minimum hordein (0.13%) was detected in kabuli type Noor-2013, followed by desi-type Wild Hybrid-16 with a value of 0.33%, while maximum hordein (11%) was found in NIAB-CH104 (Desi). In the previous study on chickpeas, a small fraction (3–7%) of prolamine was detected, but in chickpeas, they are not well characterized (63). Glutelins are a class of prolamine proteins present in the endosperm of certain seeds, mostly soluble in alkali detergent or dilute acids, and in the presence of reducing or chaotropic agents. They constitute the most important component of the protein composite collectively designated as gluten (64). Current results revealed that the fewest glutelins (0.33%) were found in Wild Hbrid-9 (desi), while CH16/06 (desi) depicted the highest (20.33%) glutelins. Earlier it was observed that chickpea seeds contained 19–25% glutelins; they are nutritional imperatives containing higher levels of cysteine and methionine than globulins (50). Consumption of gluten-containing foods such as cereals causes CD. CD is an immune-mediated disease that damages the villi that are responsible for nutrient absorption. There are only a few gluten-free food products available on the market that are more expensive (65). So the chickpea genotype Wild Hbrid-9, containing the lowest value of glutelin (3.33 ± 0.67 mg/g s. wt.), could be a promising cultivar for the food industry. The quantity of soluble sugar is an essential physiological trait that affects seed production and its cooking quality and plays an important function in abiotic stress tolerance in seeds and in storability (66). TSSs in chickpea seed was detected with the highest value (10.26%) in CM1051/11 (desi), and the least value (7.79%) was detected in the Paidar-91 desi type. Several studies reported different fractions of TSS in chickpea seeds. Goñi et al. (67) found 9.33% TSS, while Sánchez-Mata et al. (68) found 5.89 to 8.21% TSS in chickpeas. Present findings exhibited results within the previously studied range. Another study showed that the DBGV-165 genotype depicted 11.10 mg/100 g of total sugars (69). A higher concentration of approximately 17% was reported in a previous study (70). Reduced sugars are simple sugars that can oxidize other compounds. Reducing sugars are vital in central metabolic pathways, facilitating the production of secondary metabolites that boost the medicinal properties of plants (71). The highest reducing sugars were detected in Punjab-2000 (desi type) with a value of 1.733%, while the lowest value of 0.359% was found in Wild Hybrid-1. In the literature, the range of reducing sugars in chickpeas seeds was varied from 0.86 to 2.37%, and the present results are in accordance with the previous findings (72). Another finding exhibited a lower value of 0.158% reducing sugars in chickpea variety DIBG-201 (69). In the present study, the range for non-reducing sugars was given from 6.61 to 9.53%; however, the maximum value was detected in NIAB-CH2016, and the least content was observed in Paidar-91 both were of desi type. The literature indicates a lower range of non-reducing sugars in chickpea varieties, ranging from 1.3% to 3.3% (72). Another study on three Pakistani chickpea varieties reported a relatively low (3.30%) amount of non-reducing sugars than the present results (73). Starch, a major storage carbohydrate, is primarily composed of amylose and amylopectin, both of which have α-1,4- and α-1,6-linked glucose units (74). Legumes are not only an affordable source of protein but also starch, which has the advantage of being resistant to starch compared with cereal, root, and tuber starch (75). The present study depicted that chickpea seed starch content ranged from 14.13 to 83.69%, and the highest starch was detected in the kabuli type (CH55/09) and the least starch was found in the desi type (Wild Hybrid-6). Earlier studies reported that starch content in chickpea seeds ranged from 37.5 to 50.8% (76). Chickpea seeds contain higher amounts of resistant starch and amylose. In the small intestine, chickpea starch is more resistant to digestion, resulting in a lower availability of glucose, which reduces the demand for insulin by slowing glucose entry into the bloodstream (77). Resistant starch-rich diets offer numerous health benefits, including colon cancer prevention, reduced coronary cases, weight management, healthy colon, and type 2 diabetes management (78).

Anti-nutritional factors, also known as secondary metabolites, are bioactive compounds that can lower the nutritional value of plants or plant derivatives that are often utilized in human diet or animal feed (79). Anti-nutritional factors are biologically deleterious substances present in the diet that behave antagonistically to one or multiple nutrients, reducing bioavailability (80). This is generally conducted via complex formation, which diminishes nutrient absorption, leading to impaired metabolic performance and gastrointestinal functions (81). PA, tannins, gossypol, raffinose, enzyme inhibitors, saponins, lectins, goitrogens, glucosinolates, oxalic acid, alkaloids, erucic acid, hydrogen cyanide (HCN), and *β*-N-oxalyl amino alanine (BOAA) are prominent anti-nutritional agents found in food (82). Phytate is found in all grain legumes, while tannins are primarily condensed in dark-seeded grains (83). Tannins are polyphenolic compounds present in food legumes. Tannins have the ability to interact with enzymes, proteins, non-enzymes, hampering protein solubility, digestibility, and amino acid accessibility (31, 84). This study revealed tannins in chickpea seeds ranged from 5,425 to 13,775 uM/g s. wt.; however, in the high category out of 10 genotypes, 8 were of desi type, and maximum tannins were detected in Wild Hybrid-3 (desi type), which showed that in the desi type more tannins are present as compared to the kabuli type. A previous report suggested that desi chickpeas have higher tannins compared to the kabuli type because most of the tannins are condensed in dark-colored seed coats (85). Tannins, which are concentrated in the dark-colored and rough seed coat of desi chickpeas, contribute to their astringent taste, which is why smooth surface kabuli chickpeas are preferred in some regions; similar results were observed in the present study, in which desi genotypes had higher tannins than kabuli genotypes (86, 87). Some domestic processing, such as pressure cooking and roasting, significantly reduced the tannin contents in chickpeas (88).

PA, also known as inositol hexaphosphate, is a bioactive sugar molecule featuring a simple ring structure with six phosphate groups attached to each carbon atom. Pulse, cereals, nuts, and oilseeds contain substantial amounts of PA (89). The presence of anti-nutrient PA in legumes hinders the bioavailability of proteins and absorption of trace elements and macro elements such as Ca, Zn, Mg, and Fe (90–92). PA is a proton contributor that generates “phytate” anion and hydrogen ions (H^+^) that quickly form salts with available proteins and minerals (93). Furthermore, it has also been studied that the existence of PA restrains micronutrient fortification approaches because it can hamper the fortified micronutrients, leading to ineffectiveness (94).

The present study depicted PA in chickpeas ranging from 0.18 to 6.42 mg/g s. wt., and the maximum value was noticed in the kabuli genotype Tamaman-13; however, the least value was observed in the desi genotype Bhakhar-2011. It was noticed that in the low category, all the genotypes belong to the desi type, so the small seed contained a low PA concentration compared to the large-seeded. In a previous report, a considerably higher concentration of approximately 11.16 mg/g of PA was detected in chickpea seeds (95). Another study suggested that in comparison with other legumes, chickpeas contained relatively low PA and desi biotypes exhibited lower concentrations (96). Another study reported a relatively higher PA range (5.95 to 9.09 mg/g) in various chickpea cultivars (97).

Results of PCA revealed the diversity of genotypes for the traits under study. It was noticed that genotypes CH35/10, ILWC-247, Wild Hybrid-15, CH1051/11, ICCV-96030, CH56/09, etc. represented high positive factor scores, while Noor 2009, CH16/06, Wild Hybrid-4, Paidar-91, Lawaghar, etc. represented negative factor scores in PC-1. For PC-II, genotypes such as CM-88, Parbat 98, C-44, CH16/06, NIAB-CH2016, ICCV-98030, and CC98/99 have high positive scores. In contrast, genotypes including CH28/07, Paidar-91, CH28/07, WH-4, WH-9, and Noor-91 display negative factor scores. According to the results of PCA, genotypes with high positive factor scores in PC-I can be identified as promising genotypes for nutritional traits with high positive scores such as TSPs, CPs, TSSs, non-reducing sugars, reducing sugars, globulins, starch, and TFAs. Conversely, genotypes with high negative factor scores in PC-I are considered less nutritionally rich. In the case of PC-II, genotypes with high positive scores for key nutritional traits such as non-reducing sugars, TSSs, globulins, and salt-soluble proteins are identified as promising. These genotypes also exhibit lower levels of anti-nutritional factors, as both tannins and PA have negative factor scores in PC-II. This analysis provides valuable insights for selecting and breeding chickpea varieties with optimal nutritional profiles, enabling the development of varieties that are both nutritionally rich and low in anti-nutritional factors. The cluster analysis divided the 90 genotypes (desi and kabuli) into three distinct groups. Cluster I comprised the genotypes with high average values for CPs, albumin, hordein, and glutelin. These genotypes are important for breeding programs focused on enhancing protein content and quality in chickpeas. In contrast, Cluster II is composed of the genotypes with high mean values of TSPs, TSSs, non-reducing sugars, globulins, salt-soluble proteins, starch, and TFAs. These traits are crucial for improving the overall nutritional profile of chickpeas, making them more beneficial for human consumption. However, Cluster III contains genotypes with high mean values of anti-nutritional factors such as tannins, reducing sugars, and PA. These anti-nutritional factors can interfere with the absorption of essential nutrients. Identifying these genotypes helps in breeding programs aimed at reducing anti-nutritional factors. Identified chickpea genotypes through different analyses will serve as essential stable donors in chickpea biofortification strategies. They will also facilitate the expansion of biofortified crop cultivation, contributing significantly to health and nutritional security in developing countries.

## Conclusion

The present study revealed that both desi and kabuli genotypes represented vast genetic diversity for different seed nutritive and anti-nutritive traits. Genotypes with maximum TSPs, CPs, reducing sugars (Punjab-2000: D), TFAs (Wild Hybrid-15: D), albumins (Sheenghar-2000: D), globulins (ICCV-96030: D), salt-soluble proteins (ILWC-247: D), TSSs (CM1051/11: D), non-reducing sugars (NIAB-CH2016: D), starch content (CH55/09:K), and the genotypes with a minimum value of anti-nutritional constituents, i.e., glutelin (Wild Hybrid-9: D), hordein (Noor-2013: K), tannins (Wild Hybrid-1: D), and PA (Bhakhar-2011: D) are the promising cultivars for improving seed quality attributes. The findings of this study could also be helpful in addressing malnutrition-related life-threatening challenges. However, seed quality traits can be significantly influenced by environmental factors, so testing genotypes under diverse climatic conditions will ensure that the findings are robust and reliable, enhancing their practical utility in breeding programs, the food industry, and efforts to combat malnutrition. Additionally, exploring the genetic basis of these traits could uncover new genes and pathways that could be targeted in future breeding programs.

## Data Availability

The original contributions presented in the study are included in the article/Supplementary material, further inquiries can be directed to the corresponding author.
