# Supplementary material for: Demystifying the nutritional and anti-nutritional genetic divergence of Pakistani chickpea (Cicer arietinum L.) genetic resource via multivariate approaches
Source: Front Nutr. 2024 Oct 4;11:1407096. doi: 10.3389/fnut.2024.1407096 (PMC11494965; doi:10.3389/fnut.2024.1407096)
Supplement: Supplementary file 1 [file Data_Sheet_1.docx]

Figure S1: Comparison of seed protein content in chickpea genotypes (mean value ± SD). Means with different alphabets are significantly different (Tukey’s HSD, p˂0.05).

Figure S2: Comparison of seed crude protein content in chickpea genotypes (mean value ± SD). Means with different alphabets are significantly different (Tukey’s HSD, p˂0.05).

Figure S3: Comparison of seed Total Free Amino acids in chickpea genotypes (mean value ± SD). Means with different alphabets are significantly different (Tukey’s HSD, p˂0.05).

Figure S4: Comparison of seed Albumin content in chickpea genotypes (mean value ± SD). Means with different alphabets are significantly different (Tukey’s HSD, p˂0.05

Figure S5: Comparison of seed Globulin content in chickpea genotypes (mean value ± SD). Means with different alphabets are significantly different (Tukey’s HSD, p˂0.05).

Figure S6: Comparison of seed Salt soluble protein content in chickpea genotypes (mean value ± SD). Means with different alphabets are significantly different (Tukey’s HSD, p˂0.05).

Figure S7: Comparison of seed Hordein content in chickpea genotypes (mean value ± SD). Means with different alphabets are significantly different (Tukey’s HSD, p˂0.05).

Figure S8: Comparison of seed Glutelin content in chickpea genotypes (mean value ± SD). Means with different alphabets are significantly different (Tukey’s HSD, p˂0.05).

Figure S9: Comparison of seed Total soluble sugars in chickpea genotypes (mean value ± SD). Means with different alphabets are significantly different (Tukey’s HSD, p˂0.05).

Figure S10: Comparison of seed Non-reducing sugars in chickpea genotypes (mean value ± SD). Means with different alphabets are significantly different (Tukey’s HSD, p˂0.05).

Figure S11: Comparison of seed Reducing Sugars in chickpea genotypes (mean value ± SD). Means with different alphabets are significantly different (Tukey’s HSD, p˂0.05).

Figure S12: Comparison of seed Starch content in chickpea genotypes (mean value ± SD). Means with different alphabets are significantly different (Tukey’s HSD, p˂0.05).

Figure S13 :Comparison of seed Tannins content in chickpea genotypes (mean value ± SD). Means with different alphabets are significantly different (Tukey’s HSD, p˂0.05).

Figure S14: Comparison of seed Phytic acid in chickpea genotypes (mean value ± SD). Means with different alphabets are significantly different (Tukey’s HSD, p˂0.05).

**Table S1: Principal component analysis for seed nutritional and anti-nutritional attributes chickpea genotypes**

|  | **PC-I** | **PC-II** | **PC-III** | **PC-IV** | **PC-V** | **PC-VI** | **PC-VII** | **PC-VIII** | **PC-IX** | **PC-X** | **PC-XI** | **PC-XII** | **PC-XIII** |
| --- | --- | --- | --- | --- | --- | --- | --- | --- | --- | --- | --- | --- | --- |
| **Eigenvalue** | **2.917** | **1.655** | **1.435** | **1.333** | **1.152** | **1.029** | **0.893** | **0.835** | **0.766** | **0.688** | **0.546** | **0.433** | **0.318** |
| Variability (%) | 20.836 | 11.825 | 10.249 | 9.523 | 8.225 | 7.352 | 6.376 | 5.962 | 5.471 | 4.916 | 3.898 | 3.094 | 2.273 |
| Cumulative % | 20.836 | 32.661 | 42.910 | 52.432 | 60.658 | 68.010 | 74.386 | 80.348 | 85.819 | 90.735 | 94.633 | 97.727 | 100.000 |
| **Factor loadings** | **PC-I** | **PC-II** | **PC-III** | **PC-IV** | **PC-V** | **PC-VI** | **PC-VII** | **PC-VIII** | **PC-IX** | **PC-X** | **PC-XI** | **PC-XII** | **PC-XIII** |
| Tannins | 0.003 | -0.390 | 0.141 | 0.471 | -0.202 | -0.224 | 0.540 | 0.043 | -0.383 | 0.215 | 0.161 | 0.034 | 0.045 |
| total soluble protein | 0.142 | -0.301 | 0.231 | 0.291 | 0.636 | -0.125 | -0.215 | -0.258 | 0.176 | 0.403 | -0.019 | 0.132 | -0.084 |
| crude proteins | 0.124 | -0.111 | 0.217 | -0.257 | 0.144 | 0.839 | 0.260 | -0.144 | -0.028 | 0.135 | 0.087 | 0.005 | 0.139 |
| Total soluble sugars | 0.859 | 0.299 | -0.092 | 0.170 | 0.142 | 0.048 | -0.087 | 0.024 | -0.147 | -0.033 | 0.221 | -0.172 | -0.067 |
| Non reducing Sugars | 0.577 | 0.733 | -0.169 | 0.135 | 0.164 | -0.050 | 0.048 | -0.030 | -0.097 | 0.119 | 0.140 | -0.058 | 0.064 |
| Reducing Sugars | 0.501 | -0.687 | 0.120 | 0.065 | -0.029 | 0.160 | -0.223 | 0.089 | -0.088 | -0.249 | 0.143 | -0.193 | -0.215 |
| Albumins | -0.038 | 0.023 | -0.457 | 0.626 | 0.187 | 0.301 | 0.079 | -0.152 | -0.158 | -0.285 | -0.355 | 0.092 | -0.044 |
| Globulins | 0.261 | 0.218 | 0.515 | 0.228 | 0.120 | 0.004 | 0.423 | 0.314 | 0.464 | -0.205 | -0.070 | 0.029 | -0.114 |
| Salt soluble Proteins | -0.007 | 0.291 | 0.693 | -0.221 | 0.026 | -0.112 | 0.023 | -0.312 | -0.391 | -0.071 | -0.293 | -0.128 | -0.120 |
| Hordein | -0.170 | 0.235 | 0.338 | 0.366 | -0.274 | 0.300 | -0.420 | 0.468 | -0.162 | 0.252 | -0.088 | 0.099 | -0.017 |
| Gluten | -0.688 | 0.251 | 0.128 | -0.001 | 0.232 | 0.057 | -0.037 | -0.054 | -0.181 | -0.276 | 0.410 | 0.290 | -0.157 |
| Phytic acid | 0.362 | -0.100 | -0.291 | -0.527 | 0.322 | -0.043 | 0.175 | 0.427 | -0.262 | 0.116 | -0.173 | 0.203 | -0.158 |
| Starch | 0.729 | -0.183 | 0.292 | -0.026 | 0.004 | -0.154 | -0.181 | -0.006 | -0.085 | -0.291 | -0.023 | 0.315 | 0.316 |
| Total Free Amino acid | 0.570 | 0.090 | -0.094 | -0.059 | -0.596 | 0.085 | 0.036 | -0.336 | 0.143 | 0.132 | -0.001 | 0.291 | -0.237 |
| Type-Desi | -0.072 | -0.065 | 0.006 | 0.054 | 0.080 | 0.352 | 0.162 | -0.128 | 0.145 | 0.079 | 0.015 | -0.063 | 0.041 |
| Type-kabuli | 0.072 | 0.065 | -0.006 | -0.054 | -0.080 | -0.352 | -0.162 | 0.128 | -0.145 | -0.079 | -0.015 | 0.063 | -0.041 |

**Table S2: Correlation matrix (Pearson) of Seed Nutritional and Anti-Nutritional Attributes**

| **Variables** | **Tannins** | **total soluble protein** | **crude proteins** | **Total soluble sugars** | **Non-reducing- Sugars** | **Reducing Sugars** | **Albumins** | **Globulins** | **Salt soluble Proteins** | **Hordein** | **Gluten** | **Phytic acid** | **Starch** | **TFA** |
| --- | --- | --- | --- | --- | --- | --- | --- | --- | --- | --- | --- | --- | --- | --- |
| Tannins | **1** |  |  |  |  |  |  |  |  |  |  |  |  |  |
| total soluble protein | 0.077 | **1** |  |  |  |  |  |  |  |  |  |  |  |  |
| crude proteins | -0.070 | 0.032 | **1** |  |  |  |  |  |  |  |  |  |  |  |
| Total soluble sugars | -0.056 | 0.096 | 0.053 | **1** |  |  |  |  |  |  |  |  |  |  |
| Non reducing Sugars | -0.155 | -0.015 | -0.042 | **0.816** | **1** |  |  |  |  |  |  |  |  |  |
| Reducing Sugars | 0.158 | 0.185 | 0.157 | **0.351** | **-0.256** | **1** |  |  |  |  |  |  |  |  |
| Albumins | 0.097 | 0.048 | -0.016 | 0.094 | 0.104 | -0.011 | **1** |  |  |  |  |  |  |  |
| Globulins | 0.076 | 0.074 | 0.084 | 0.194 | 0.196 | 0.007 | -0.070 | **1** |  |  |  |  |  |  |
| Salt soluble Proteins | -0.023 | 0.015 | 0.093 | -0.006 | 0.071 | -0.125 | **-0.248** | 0.146 | **1** |  |  |  |  |  |
| Hordein | 0.015 | -0.063 | -0.003 | -0.041 | 0.000 | -0.067 | 0.016 | 0.086 | 0.086 | **1** |  |  |  |  |
| Gluten | -0.086 | -0.081 | -0.023 | **-0.403** | -0.184 | **-0.375** | 0.034 | -0.093 | 0.134 | 0.118 | **1** |  |  |  |
| Phytic acid | -0.094 | -0.032 | 0.109 | **0.229** | 0.157 | 0.128 | -0.121 | -0.061 | -0.094 | **-0.241** | **-0.241** | **1** |  |  |
| Starch | 0.029 | 0.164 | 0.013 | **0.491** | 0.206 | **0.488** | -0.121 | **0.212** | 0.148 | -0.083 | **-0.384** | 0.191 | **1** |  |
| Total Free Amino acid | 0.006 | -0.159 | 0.082 | **0.364** | **0.282** | 0.152 | -0.067 | 0.020 | -0.023 | -0.069 | **-0.439** | -0.002 | **0.320** | **1** |
| *Values in bold are different from 0 with a significance level of alpha=0.05* | | | | | | | | | | | | | | |
